# Ten simple rules for forming a scientific professional society

**DOI:** 10.1371/journal.pcbi.1005226

**Published:** 2017-03-23

**Authors:** Bruno A. Gaëta, Javier De Las Rivas, Paul Horton, Pieter Meysman, Nicola Mulder, Paolo Romano, Lonnie Welch

**Affiliations:** 1 School of Computer Science and Engineering, The University of New South Wales, Sydney, Australia; 2 Bioinformatics and Functional Genomics Group, Cancer Research Center (CiC-IBMCC, CSIC/USAL/IBSAL), Salamanca, Spain; 3 Artificial Intelligence Research Center, AIST, Tokyo, Japan; 4 Biomedical Informatics Research Center Antwerp (biomina), University of Antwerp/Antwerp University Hospital, Antwerp, Belgium; 5 Department of Mathematics and Computer Sciences, University of Antwerp, Antwerp, Belgium; 6 Computational Biology Division, Department of Integrative Biomedical Sciences, IDM, Faculty of Health Sciences, University of Cape Town, Cape Town, South Africa; 7 Bioinformatics group, Proteomics laboratory, IRCCS AOU San Martino IST, Genoa, Italy; 8 School of Electrical Engineering and Computer Science, Ohio University, Athens, Ohio, United States of America

In this article, we present ten simple rules to assist in the formation and management of a scientific professional society. We define a "scientific professional society" (also often referred to as a “scholarly society”) as an association of people who come together to promote progress in a specific technological or scientific area and to facilitate the interaction of interested people on a regional, national, or international level. For the rest of this article, we will use the term “society” to refer to a scientific professional society.

Some emerging disciplines in biology where scientific professional societies have appeared relatively recently include computational biology and bioinformatics, systems biology, and functional genomics. The main professional body representing computational biology is the International Society for Computational Biology (ISCB) [1], founded in 1997. For systems biology, the International Society for Systems Biology (ISSB) [2] started as an International Conference created by Hiroaki Kitano in 2000, which led to the formation of the society. In the field of functional genomics, the Functional Genomics Data Society (FGED) [3] was formed in 1999 as the Microarray Gene Expression Data Society (MGED) in recognition of the need to establish standards for sharing and storing genomic data. MGED changed its name in 2010 to FGED. These examples show that the formation of a new scientific professional society is often triggered by the emergence of a new scientific discipline or by technological developments in science. This, of course, is not the only reason for developing a new society. Some computational biology-related societies with a very targeted focus include the International Society for Biocuration (ISB) [4,5], which is aimed at ensuring the best possible annotation methods and tools for curation of biological databases, and the Global Organization for Bioinformatics Learning, Education and Training (GOBLET) [6,7], whose aim is the development of the best possible educational strategies and materials in bioinformatics.

However, the majority of societies aim to foster networking in a narrower geographical area, such as a city or a country, and these are the focus of this article. The rules presented draw on the collective experience of the authors, who all have been active in the formation and/or development of computational biology societies in their respective regions and countries in Asia [8,9], Africa [10], North America [11], Europe [12], and Ibero-America [13]. These “rules” are by no means prescriptive but are meant to act as guidelines and suggestions to help avoid some common pitfalls.

## Rule 1: Have a clear scope and vision

Setting up a new society will require a lot of work—it is therefore a good idea, before getting started, to identify the goals and the target audience of your society, as these will have a strong impact on the process. A society’s goals can include improving the profile of a scientific discipline through public advocacy and government representation, and fostering networking, information sharing, mentoring, career opportunities, leadership training, and professional development. To couch this process in strategic management terms [14], identifying the "vision" of the society (its purpose and aspirations) is important to guide your "strategy" (the practical steps to achieve the vision), which in turn determines the processes and resources required. For example, a society aiming to run a large annual conference will require setting up significant infrastructure to manage budget and liability, in contrast to a society whose goal is to run a monthly seminar series in a local university and can get by with a mailing list and a small cash pool for drinks and snacks.

The questions you need to ask yourself at this stage are similar to those a new business needs to address: what "products" or "services" are you going to offer? These could include conferences, seminars, publications, networking events, student scholarships, and public advocacy. Who are your intended customers in terms of scientific discipline, geographic region, and career stage? What “benefits” is your society going to provide to these members? Who is your “competition”: other local societies or larger regional/national/international societies covering similar or related scientific domains? And how will your proposed society differentiate itself from these so that it will attract and retain members? If other societies exist in related scientific areas, you need to make sure you offer something unique, but at the same time, look for opportunities to dynamically partner with other societies, for example for joint meetings (see Rules 2 and 9). While you do not want to be delayed by over-planning, it is important to think about these big questions in order to start on the right foot and ensure your resources are spent productively and, more importantly, that you really want to commit your time and energy to a new society instead of an already existing group [15].

The answers to these questions will form the business plan of your society, and like any business plan, they will need to be revisited at regular intervals (for example, every two years) as the society and its landscape evolve.

## Rule 2: Start an annual meeting

An annual gathering, such as a conference [16] or a workshop [17,18], will propel you forward as you build a community of individuals with a common interest. The meeting will provide a focus for your interactions and will provide the opportunity to interact regularly throughout the year as you plan the event. An annual in-person meeting provides the opportunity to build relationships and to have spontaneous ideas for enhancing the offerings of your community.

When planning the meeting, form an organizing committee that spans the geographic region and that includes diverse perspectives (e.g., public and private universities, government research facilities, medical research labs, and industry). Encourage members to seek buy-in from their home institutions in the form of co-sponsorship for the event. Among the invited keynote speakers, include some people from the local region. If possible, try to partner with an international society in the same area (for example, ISCB for computational biology) to tap into their meeting organization expertise and to help with event promotion. Finally, start small, and make incremental improvements to the meeting each year.

## Rule 3: Make history: Document everything

You may have just started, but the years will go by quickly, and (documented) tradition lends credibility. So spend a moment early on to consider how you will document the activity of your society and the contribution of your members to your society and the research community at large. Plan to archive the webpages of all of your society’s major events, especially your annual meetings, starting from the first one! A few years down the road, consider starting some awards, which may grow to become prestigious in the future.

Internal documentation is also essential, both for transparency of decision-making and succession planning as the society changes over time and new officers come on board. Archive all meeting minutes and, more importantly, all contact information, including that of officers and sponsors, using a collaborative online platform that can be accessed readily by current and new society officers.

## Rule 4: Start small or build on existing scientific and professional networks

A professional society cannot be fully designed by a single scientist alone: it requires consensus, as well as exchange of ideas, expertise, and skills. Required skills are very different from research expertise and, without suitable mentoring, can only be acquired by (many) trials and (some) errors. If at all possible, try to take advantage of the experience available in existing societies and networks in your area. The benefits are both practical (for example, lists of interested parties and service providers) and conceptual, including ways of sharing ideas and finding consensus.

In most cases, starting with a small number of resolute members is more efficient than trying to be all-encompassing from the start. It makes it easier to collaborate and share a common vision, assign duties, and make consensus decisions. Starting small, however, does not mean that you should remain small. A strategy for growing regularly by adding new members or involving more institutions must also be defined (for example, through scientific events that grow larger from year to year, involve a range of participants, and are located where potential members are).

## Rule 5: Set up your governance structure and constitution

Formalising the structure of the society is an essential step towards a working association. The task may seem daunting at first, so it is best to start with a simple set of terms of reference and by-laws and grow from there as the society expands. You may want to seek inspiration in the constitution and articles of governance of existing societies, many of which are directly available from their website: see, for example, the by-laws of ISCB [19] or of the American Society for Human Genetics [20]. One generally successful approach is to choose an initial executive committee made up of committed volunteers, with, if at all possible, an emphasis on inclusivity and diversity, including geographic and gender diversity. A typical executive committee structure includes a president, a secretary whose role is to keep records and minutes, a treasurer who focuses on the all-important financial aspects, and a few vice presidents with a focus on specific missions of the society (education, conferences, communications, industry outreach). A smaller group is more efficient; however, it is essential that the duties of the society are not left on the shoulders of one or two people.

The executive committee can then formulate an initial set of by-laws covering executive and committee terms and election procedures, as well as create relevant subcommittees and define their terms of reference. An emphasis on diversity, including gender and career stage diversity, is important at this stage to ensure the leadership is representative of its members. Organisation management is not a standard scientific skill, so don’t plan to do everything yourself, and be ready to seek advice. As the society (and its finances) grow, hiring a dedicated executive professional may become necessary.

## Rule 6: Be inclusive

Starting a society with some of your closest contacts and frequent collaborators is easy but can limit the growth potential of your new group, as you will tend to have similar contacts and travel in the same circles. It is therefore critical to think beyond your partners in research and reach out to those whom you might not have had reason to contact before. Try not to exclude anyone who wants to get involved, especially early on. Furthermore, especially for organisations with a regional focus, it is important to be inclusive and span the breadth of your domain at the start. Your outreach will be greatly aided by including individuals from different universities and institutions. For example, each institution has their own procedures for mass mailing, and some might have valuable funding or sponsorship programs. Having someone on the inside can help to navigate these procedures. Reaching out to these people can be as simple as talking to them during conferences and discussing your plans during one of the breaks. Sending a personalised email or making a call to potential partners about your intentions to form a new society can sometimes yield surprising results. If they themselves might not be interested, they often know someone who might be. Inclusiveness is also essential for accountability. The society should not be perceived by outsiders as a closed shop or an exclusive club but should strive to be open and act for the benefit of the community, rather than a select few.

Once the society is up and running, it is critical for its longevity that new people can still become involved easily. A web form or contact email on the website may seem impersonal, but it can be very useful to direct interested parties. Speak to people at different events and get them excited about the planned activities and the society as a whole. In addition, always be on the lookout for individuals who might be affiliated with institutions that are currently not represented.

Many successful societies have a student-focused component aimed at PhD students and other junior scientists, often run by the students themselves [21]. This can take the form of local student-run chapters, dedicated student seminars/workshops, and even local journal clubs [22]. Creation of a solid support structure, along with finding a motivated group of students to get things started, is critical for the development of such student-run activities. Simply providing access to the infrastructure that has been set up for the society, such as mailing lists, web space, or even bank accounts, will facilitate their start-up. It is important to not just assign tasks to the students, however tempting it may be to use them as cheap labor, but to let them develop their own plans and activities within the society: they will be more motivated to help out, and this will increase their sense of belonging to the group. In addition, for many, this is a very useful learning experience with regards to many different kinds of “soft skills” (such as organisation, communication, and leadership) that are often missing from a typical PhD program [23]. The students of the society may one day be its leaders, so it is important to show what such a society can do and mean for them from the start and to include student representatives in the leadership of the main body of the society.

## Rule 7: Plan the financial and legal aspects

If you want your society to be more than an informal, volunteer-run effort, it is going to require funding. The most common funding sources for societies are membership fees and conference proceeds, but you should also investigate what grants and funding schemes are available in your region that your society could leverage. In many countries, state, institutional, and philanthropic grants are available for purposes such as improving communication and networking between scientists, strengthening a particular scientific discipline, or meeting professional education needs. Such grants can provide a great source of funding for one-off projects or for seeding a new initiative and are often easier to obtain than major research grants. They are, however, not always well-publicised, and it’s a good idea to set up a fundraising taskforce with members from a wide range of institutions that keeps an eye out for funding opportunities. Diversity is key, as taskforce members from different circles will be more likely to hear about grants and sponsorship opportunities from different sources. The key to many succesful societies is their ability to leverage grants supporting conferences and workshops [24,25] and networking/connection [26,27] for such purposes.

However, these grants are typically one-off, and more sustainable sources of funding are also necessary to provide for longer term planning and continuing growth. In this context, membership fees can be a double-edged sword, as they require a whole infrastructure to collect and administer, and more importantly, they will act as a deterrent for potential members. You should consider carefully whether the income from membership fees is absolutely necessary and whether your society offers sufficient value for its fees to attract and retain members.

Conferences, on the other hand, can be both a great way to meet some of your society’s goals and a way to raise some income, but they do require a lot of commitment and work (see Rule 2). They also require a substantial starting investment to get underway.

In any case, if your society is going to collect and manage money, it will need an appropriate legal structure that will provide confidence to its members, as well as protect its officers from financial and legal liability. The exact requirements for this vary from country to country, and you need to familiarise yourself with your local laws and structures. Some countries will require the society to become incorporated as a nonprofit organisation, while others may allow a larger “parent” institution to manage the accounts to keep administrative overheads to a minimum and provide some legal “umbrella.” For example, GOBLET and the European Molecular Biology network (EMBnet) [28] make use of the Dutch “Stichting” (private foundation) legal structure, while ISCB is incorporated as a nonprofit, tax-exempt corporation in the state of Delaware. These legal details can be difficult to navigate for scientists; however, many institutions have an in-house legal department that can provide guidance and some valuable advice. If your society is going to span multiple countries/states, it is also worthwhile to investigate which of these countries/states offers the most convenient laws and taxation regimes for your purposes.

## Rule 8: Design a clear communication strategy

Communication is key to a successful society. Members need to feel like they are part of an active community and kept informed of relevant activities. A professional society website is important as the main source of information on the society’s aims, governance, events, and membership information. A yearly event calendar is a relatively low-maintenance resource that can draw existing and potential members to the website and advertise the society’s events. The society should decide whether these sites will be open to the public or restricted to its members. Technically, there are a number of frameworks on which to build a professional-looking website that also allow you to keep track of your membership. These include the open source Drupal [29] and Wordpress [30] content management systems, which are nowadays preinstalled on many web hosts. A mailing list is essential for reaching all members, and one should have a mechanism in place to keep the list up to date as memberships lapse or are renewed. This list should be active with regular summaries of activities to show that the society is alive and keen to involve its members. Joining a society and then not hearing from them until renewal time does not provide an incentive to renew.

These formal mechanisms need to be complemented by an appropriate social media strategy that allows not only the society to communicate with members but also members to communicate with each other. Excellent strategies for the development of your online outreach have been proposed by Bik et al. [31] The goal should be to have a presence on the sites where the members are, which can vary from country to country. Facebook [32] has the highest user base; however, for many users, it is restricted to personal rather than professional contacts. LinkedIn [33] and Yammer [34] are focused on professional networking but have smaller user bases. Twitter [35] is a convenient avenue for short communications and fostering a sense of community. Sites such as Meetup [36] and EventBrite [37] are also useful for building social networks based around events. A Meetup group can even be used as a simple membership database and mailing list manager if you are not collecting membership fees. However, as with the mailing list, new content must be added regularly in order for the site (and, by extension, the society) to be perceived as active. Remember that community participation on social media can sometimes backfire, and suitable posting policies and active moderation should be put in place to avoid inappropriate postings that could tarnish the society’s image.

It is also important to develop your branding and some marketing materials, such as brochures and posters that can be displayed at conferences to inform members about the society and to attract new members. Hosting an exhibition stand at conferences attracts interest but may be expensive. An alternative is a poster within the conference poster session. You could involve younger members of the community in the development of the branding and of marketing materials (for example, through competitions for logo designs or free membership for the brochure designer). The tone of the brochure should reflect the audience for which it is intended (slightly less formal and more fun for students and academics versus formal and professional for attracting industry partners). Marketing can also be achieved through publications in other organisations’ newsletters (for example, ISCB and EMBnet for computational biology) or through regular society newsletters. Whichever mechanism is used for communication, make sure it is active to keep the members engaged and interested.

Internal communication between society officers also needs to be considered. Regular face-to-face meetings tend to be most efficient, but if that is not possible, a number of free teleconference options are available [38,39], provided the teleconference involves only a few participants. Some officers may also have access to commercial solutions through their institutions. Websites such as Doodle [40] and timeanddate [41] are useful for setting up meeting times, especially when the participants are in different time zones. Remember to keep good records of all the meetings (Rule 3).

## Rule 9: Seek partnerships and affiliations with other societies

All scientific societies have a goal of improving networking and communication in their areas, and in that context, significant synergies can be generated by partnering with more established societies. Benefits will include access to expertise and, often, to services including conference organisation (see Rule 2) and legal (see Rule 7) and administrative support. Any society whose goals include advocacy for its scientific discipline will gain a lot of traction by being able to call on the strength of a larger group. Your society may also be able to access some specific funding schemes and grants through partnership with a larger organisation.

For example, within the discipline of computational biology, it is valuable to partner with ISCB, which has in place a comprehensive affiliation scheme to support and collaborate with other bioinformatics societies. Groups that serve a particular geographic region can become regional affiliates of ISCB, while topic-based groups can align with ISCB as communities of special interests (COSIs). ISCB offers assistance to groups that are in the formative stages, provides opportunities for associations to have a global impact, and rewards groups that make significant contributions to the broader community. Connection with an international society leads to enhanced opportunities for collaboration, provides access to additional sources of expertise, makes available additional resources, and infuses additional energy.

## Rule 10: Have fun: Enjoy science and make friends

Academic societies are a serious business that promote crucial scientific collaboration. But at the same time, they are social clubs supported by volunteer time. Your society will be more sustainable if the members not only find the society’s activities meaningful but also find them to be enjoyable and special experiences sharing time with other colleagues. One easy but effective way to facilitate this is to make sure you leave some free time (for example, lunchtimes and evenings) when you schedule events to allow the participants to socialize. One of the greatest side benefits of a society is the opportunity to meet and exchange experiences with many people of different backgrounds, with returns that can go beyond the scientific. By holding a meeting (especially if it is in an interesting venue), your society will provide a catalyst for fun and productive networking and, occasionally, even the start of lifelong friendships.

## Summary

Starting a professional society is not something that should be entered into lightly: it requires work and dedication that can detract from your research projects and other career objectives [15]. It certainly should not be attempted on your own. But there are many potential benefits and rewards in terms of promoting the profile of your discipline (which, in turn, can affect your grant success), boosting your own profile, developing useful management and leadership skills, finding mentors, and forming essential contacts and partnerships, as science is becoming increasingly collaborative. A successful society will be a source of lifelong learning and new ideas, will open up career opportunities for students and investigators, and will provide a much stronger voice for your discipline than an isolated scientist.
